# Surface Tension of Liquid Organic Acids: An Artificial Neural Network Model

**DOI:** 10.3390/molecules26061636

**Published:** 2021-03-15

**Authors:** Mariano Pierantozzi, Ángel Mulero, Isidro Cachadiña

**Affiliations:** 1 Scuola di Architettura e Design, Università di Camerino, 63100 Ascoli Piceno, Italy; m.pierantozzi@univpm.it; 2 Departamento de Física Aplicada, Universidad de Extremadura, 06006 Badajoz, Spain; icacha@unex.es

**Keywords:** surface tension, organic fatty acids, carboxylic acids, aliphatic acids, polyfunctional acids, artificial neural network

## Abstract

An artificial neural network model is proposed for the surface tension of liquid organic fatty acids covering a wide temperature range. A set of 2051 data collected for 98 acids (including carboxylic, aliphatic, and polyfunctional) was considered for the training, testing, and prediction of the resulting network model. Different architectures were explored, with the final choice giving the best results, in which the input layer has the reduced temperature (temperature divided by the critical point temperature), boiling temperature, and acentric factor as an independent variable, a 41-neuron hidden layer, and an output layer consisting of one neuron. The overall absolute percentage deviation is 1.33%, and the maximum percentage deviation is 14.53%. These results constitute a major improvement over the accuracy obtained using corresponding-states correlations from the literature.

## 1. Introduction

In some industries, several physicochemical processes involving fluids require knowing how the surface tension of different liquids changes with the temperature along the vapour-liquid equilibrium. Capillarity, droplet and bubble formation, extraction, etc. are clear examples [1,2]. In particular, organic acids are present as additives in different kinds of foods and drinks, vegetable oils, medicines, fuels and biofuels, and detergents, etc. Thus, it is useful to study the behaviour of their surface tension both in their technological applications and in understanding the underlying processes that occur in natural substances [3,4,5,6,7,8]. For instance, the so-called Kelvin equation, which is used to study the curvature effects in small droplets, includes these compounds’ surface tension [9].

As well-known, the surface tension at the vapour-liquid equilibrium depends only on the temperature, decreasing as the temperature increases and reaching a zero value just at the critical point. Any model or correlation must take this behaviour into account and be based on the consideration of a populated data set. Experimental data for the surface tension of liquid organic acids are scarce, and sometimes the only values available are those predicted by applying Sugden’s method [10]. An alternative is to use one of the various more modern methods to calculate and predict those values, methods which generally are based on analytical models [3,11,12,13,14,15,16,17,18,19,20,21,22,23,24,25,26]. These analytical models include purely empirical correlations, as well as those based on the use of the corresponding-states principle or more sophisticated chemical-group-contribution methods, including quantitative-structure-property models. 

Models for the surface tension of liquid organic acids have been considered only in a few papers. In particular, the proposal of Sastri and Rao [12] merits attention, based on the use of the corresponding-states principle. It includes the normal boiling temperature, critical temperature, and critical pressure as inputs. In our opinion, the main drawback of this correlation is the very narrow temperature ranges that the authors considered, which limits its applicability. Di Nicola et al. [22] have shown that for most of the organic carboxylic acids considered, this correlation gives absolute percentage deviations greater than 10%. 

Two simple models were proposed by Chumpitaz et al. [14], in which they took into consideration their own measurements of the surface tension for dodecanoic, hexadecanoic, oleic, and tetradecanoic acids. In any case, the temperature range considered was very narrow.

Delgado and Díaz [18] evaluated the performance of their quantitative-structure-property model when applied to the surface tension at 298 K of some organic compounds. The percentage deviations for the seven carboxylic acids considered were below 2.3%. Only one source of data was used in that work, i.e., no comparison with other sources was made.

Díaz-Tovar et al. [3] proposed a group-contribution model for the surface tension for 12 organic acids. For that, they employed 46 data values obtained from the database named CAPEC. The overall absolute percentage deviation was 0.81% but, as the authors state, extrapolations could lead to inappropriate results.

Two corresponding-states based models for the surface tension of liquids were proposed by Gharagheizi et al. [17]. For that, they considered all the substances included in the DIPPR database [27] at that moment. Several families of organic acids were considered, and the deviations obtained were in the range of 10% to 30%. This is an expected result due to the high number of substances and data considered.

Di Nicola et al. [22] selected the experimental surface tension values that are available in the DIPPR database [27] for carboxylic acids. Those data were fitted using a scaled equation with three input parameters: Critical density, critical temperature, and radius of gyration. Unfortunately, no single model could reproduce all the data with good accuracy. Then, they proposed one model mainly for weak acidity fluids and another for strong acidity fluids. Overall, absolute deviations of 2.7% and 3.4% were obtained, respectively. The models’ predictive capacity was tested for five acids, in which the database contained one surface tension value, that had not been included in the model’s development. An overall absolute deviation of 3.1% was obtained for these five acids. Finally, the behaviour of the model at higher temperatures was checked using six data for ethanoic acid, obtaining percentage deviations around 3%.

Finally, Naef and Acree [26] proposed a chemical-group-contribution model for the surface tension of ordinary organic and ionic liquids. The main idea is to consider a complete breakdown of the molecules into their constituting atoms and then distinguish them by their immediate neighbour atoms and bond constitution [28]. This method was applied to an amount of 1893 liquids but only for the reference temperature of 298.15 K and considering different sources of data for each kind of liquid. Results for some organic acids were given as Supplementary Materials, where they showed that the deviations between the experimental and calculated values are small. This is an expected result due to the complexity of the proposed model and to the fact that only one surface tension data was considered for each fluid, i.e., the dependence of this property on temperature was not considered. 

Mulero et al. [23] highlighted the main limitations of the previous analytical models. In most cases, few data are used for each acid, and they are usually taken from a single source. Moreover, the temperature ranges considered are narrow. To avoid this situation, they used different sources (databases, books, and published papers), and collected and screened the available data carefully. As a result, they built a new database containing 2066 data for 99 organic fatty acids, including aliphatic, carboxylic, and polyfunctional acids. Then, they proposed empirical correlations which contain adjustable coefficients for each of the 99 organic acids considered. In particular, the analytical expression for the correlations is the same as that used in the REFPROP NIST program [29], with the inclusion of two or four adjustable coefficients for each fluid. The proposed correlations reproduce the data with individual absolute percentage deviations below 1.6% and maximum percentage deviations below 8% [23]. Despite the accuracy of the individual correlations, the coefficients have to be obtained for each fluid separately. Moreover, the input properties needed in the general correlations or models are not always available, and the analytical expression for the temperature dependence, generally consisting of a power law with a fixed exponent, limits their applicability in the sense that they cannot give good results simultaneously for both low- and high-temperature ranges [24,25]. Finally, as shown in Section 3, no general analytical model is present for the surface tension of a large number of organic acids with acceptable accuracy.

As an alternative to the previous methods, some authors have used artificial neural networks (ANNs) for the study of the surface tension and other thermophysical properties [16,30,31,32,33,34,35,36,37,38]. ANNs take their inspiration from biological neural networks, with the mathematical model of a single neuron and the way in which neurons are interconnected (i.e., the “architecture”), leading to a framework for different machine learning algorithms. In techniques of this kind, a training process lets the model “learn” how the input and output properties are connected, leading to accurate results even for those data not included in the training process.

Very recently, Faúndez et al. [38] reviewed the work on ANN models in the correlation and prediction of liquid properties, pointing out their capabilities and limitations. For the advantages, they highlighted their ability to work with incomplete information, as well as their capacity to learn and relate variables (taking care that sometimes the models mostly memorize and lose any predictive capability). For the main disadvantages, three were pointed out including: i) No specific rules for obtaining the architecture of ANNs (experience and trial and error methods are needed to solve this); ii) the time consumed by the network to find a solution is unknown; and iii) the optimum values for the weights and bias parameter could be not guaranteed.

In particular, they showed that, even in well-trained ANN models, the predictions obtained might not always be adequate. Therefore, they give various recommendations for the development, training, and analysis of the results of ANN models. Most of these recommendations have been followed here.

Many architectures can be used for ANNs, but as Mulero et al. [34,35] pointed out, the so-called feed-forward multilayer perceptron can be considered very successful in predicting the surface tension. Therefore, the latter model is proposed here.

In a multilayer perceptron, the input and output layers are fixed. The input layer contains one neuron for each property which is used as the input variable. The calculated property is given by one neuron placed in the output layer. Some hidden layers are added to connect the input and output layers. These hidden layers contain an undetermined number of neurons connected in parallel. 

The training procedure will set the bias and weight of each neuron input so that a predetermined objective error is reached. In addition, a non-linear activation function establishes the relationship between the input and output of a given neuron. The sigmoid function is used in this work, as commonly done in feed-forward networks [16,21,34,35,38].

Once the basic model has been established, the main task of the programmer is the selection of inputs or in other words, the input variables that are most likely to be significant for the surface tension, as well as the number of neurons in the hidden layers. Here, the programmer’s experience and ability are essential. Thus, it is necessary to try different architectures and analyze the results carefully [38]. It is possible to find that the model is over-fitted leading to large unacceptable errors. This can be avoided by dividing the dataset into three subsets: Training, test, and prediction [32,33,38]. While the training set is used to establish the main parameters of the model (bias, weights, etc.), the test set (sometimes called the validation set) is used to avoid overfitting, compare different ANNs, and then choose the appropriate model. Therefore, the training and test data sets are used in the runs to select the architecture of the model. Finally, a separate data set, different from the training and test sets and never used in the model’s definition, is used to evaluate if the model can predict new values [38].

Various ANN models have already been used for the surface tension of pure liquids of different kinds [16,21,30,31,34,35,36,37] and mixtures [31,39,40]. In particular, the proposals for pure liquids, published from 2008 to 2017, were summarized by Mulero et al. [34]. According to that analysis, it is clear that in most cases, only a single data source was used in the selection of the reference values. This may limit the applicability of the ANNs as it seems to be clear that, for a significant number of fluids, different sources give clearly different values for the same property at the same temperature. Moreover, Mulero et al. indicated that, as expected, the number of input properties, layers, and neurons needed in an ANN is directly related not only to the size of the data source used but also to the dispersion of the data when different sources are used. Thus, if data from different sources do not perfectly agree, this can lead to the use of more complex ANN architectures, as is the case for the ANN model proposed by Mulero et al. [35] to calculate the surface tension of 147 alcohols of different kinds, which requires four input properties and two hidden layers containing 21 neurons each.

The summary of artificial neural network models for the surface tension made by Mulero et al. [35] in Table 1 must be complemented with two recent proposals by Lashkarbolooki and Bayat [36] and Hosseini and Pierantozzi [37], respectively. In particular, Lashkarbolooki and Bayat [36] considered an ANN model applicable to 37 normal alkanes, 37 1-alkenes, and 17 cycloalkanes. The input properties required are the temperature, critical temperature, and carbon number of each fluid. All the 5461 values for the surface tension were obtained through the Van der Waals model with individual parameters for each fluid taken from the Yaws and Gabbula handbook [41]. From our perspective, the use of a correlation as a source of data may be regarded as a limitation on the validity of the model for predictions. In any case, Lashkarbolooki and Bayat [36] have shown that, at least for heptane and cyclohexane, the values used are in good agreement with those obtained from other sources. The chosen ANN architecture contains a hidden layer with 27 neurons, and the maximum deviations obtained were 0.47, 0.40, and 0.43 mN/m (no percentage deviations were given) for normal alkanes, 1-alkenes, and cycloalkanes, respectively. As the authors noted, most of the predicted data are calculated with deviations from −0.2 to 0.2 mN/m, which show the adequate accuracy of the model [36]. These small deviations, together with the requirements of only three well-known input properties as inputs, can be considered as the main advantages of this ANN.

More recently, Hosseini and Pierantozzi [37] have proposed an ANN model to calculate the surface tension of nine fatty acid esters and three biodiesels. Two hidden layers with nine neurons in each one were needed. This simple ANN was applied to a dataset containing only 137 points, obtaining an overall absolute percentage deviation of 0.44% and a percentage deviation below 4.5% for each datum. The input properties were the reduced temperature, a pseudo-molar density, and the dipole moment. As an alternative, Hosseini and Pierantozzi [37] also considered a molecular thermodynamic model, which permits the calculation of 147 surface tension values with an overall absolute percentage deviation of 1.82% when the dipole moment is taken as an adjustable parameter. Although this molecular model is not predictive (since the dipolar moments of the molecules are considered as an adjustable parameter) and gives higher percentage deviations, it has the advantages of having a theoretical molecular basis and offering values for the coexistence equilibrium densities. As a counterpart, the ANN model is purely empirical and contains a greater number of adjustable parameters, but this permits its use for predictions and obtaining lower percentage deviations.

From the above analysis, it is clear that the use of ANNs has yet to be considered in the literature for the surface tension of large numbers of organic fatty acids, over wide temperature ranges, and using different data sources.

## 2. Methodology

The dataset previously compiled by Mulero et al. [23] is considered here. It contains selected values for 99 acids belonging to the aliphatic, carboxylic, and polyfunctional families according to the classification made by DIPPR [27]. In particular, the main data sources used for that data set were the DIPPR database [27], DETHERM database [42], and three books by Wohlfarth and Wohlfarth [43,44,45]. New data were added from literature papers, and finally, for every liquid, the available data were checked before selection. Thus, some values were removed from the final dataset. Moreover, more than one value can be found at the same temperature and fluid. This clearly influences the calculation of deviations between the proposed model and the selected values at this specific temperature. All these details on the dataset, including graphical displays, are available in Mulero et al. [23] as Supplementary Materials.

Of the 99 liquids considered by Mulero et al. [23], no value for the boiling temperature is found for terephthalic acid, so for this present work only 98 acids (2051 data) were considered. These are listed in the Supplementary Materials (Table S1). The acid name, number of data, temperature range, critical point temperature, boiling temperature, and acentric factor values are given. The DIPPR database [27] was taken as the source of these last three values which are given since they are used as input properties in the ANN model. The data selected were taken as a whole by the ANN program, so the differences in the numbers of data per liquid (from 4 to 165) should not affect the final overall result.

The following deviations and statistics were calculated to analyze the accuracy of the different models. First, the percentage deviation of the model from the *i*-th point of the dataset (*PD_i_*, %) is defined as:

(1) $$PD_{i}=100\frac{\sigma \left(T_{i}\right)-\sigma_{i}}{\sigma_{i}},i=1,2,\ldots ,N$$

where *σ_i_* and *T_i_* are the surface tension and temperature, respectively, *σ(T_i_*) is the value given by each model, and *N* represents the number of data. Another useful parameter is the maximum absolute value of the *PD_i_*, denoted by PDm, which gives information on the model’s worst prediction. This PDm value is usually found near the critical temperature, where the values of *σ_i_* go to zero. Thus, in the high-temperature range, a small absolute deviation can lead to a high PD value. This fact is common for any model that could be used.

It is possible for the ANN to give good figures for the whole dataset, but a bad one for a specific fluid. Therefore, the absolute average deviation (AAD) for a fluid is defined as:

(2) $$AAD=\left(\sum_{i=1}^{N}\left|PD_{i}\right|\right)/N$$

where *N* is the number of data for that fluid.

As a step prior to the development of an ANN model, several corresponding-states models were checked. Results were obtained using the Brock-Bird [11], Sastri-Rao [12], Pitzer [13], and Gharagheizi et al. [17] models. These models were selected since they are well-known and our database contains the needed input properties, with the only exception being terephthalic acid, in which the boiling temperature is not available.

Table 1 gives the main results for these four CSP models when all the selected data of the 98 liquid acids are considered. The needed input properties, the number of fluids with the AAD value lower than 10%, the maximum and minimum AAD values, and the overall AAD value when considering the whole set of data are given. Table S2, in the Supplementary Materials, gives the AAD values obtained with these models only for those substances, in which this deviation is below 10%.

As shown in Table 1, no corresponding-states model can be used with good accuracy for the whole set of acids considered here. Indeed, the overall deviations are all greater than 20%. This was expected as these models were not specifically designed for organic fatty acids (despite some of them include several fluids of this kind). On the other hand, some of these models can be accurate for certain particular fluids, as shown in Table S2. In conclusion, a corresponding-states model can lead to very different results when used for different fluids even if they belong to the same family, so these models must be used with caution. Table S2 can serve as a reference for researchers interested in applying a model of this kind to a particular acid.

In view of the poor results obtained by previous models, we have tried to update them and make new proposals to increase their accuracy. Unfortunately, reducing the AAD by a significant amount seems to be very difficult when models of this kind are involved. This may be due to the very different behaviours of the acids considered [23] and the large amount of available data. It is clear that more complex methods are needed for obtaining significantly better results, and the ANN models presently seem to be an excellent alternative [16,21,30,31,32,33,34,35,36,37,38].

The neural network algorithm included in the Wolfram Mathematica software was used to develop the new ANN model, together with several routines implemented to choose the best network configurations. All the variables were normalized by scaling their values from zero to one, to avoid large differences between ranges in the input values and, consequently, possible saturation of the model weights.

The procedure followed takes into account the recent recommendations by Faúndez et al. [38], and is very similar to that followed in other papers [21,33,34,35]. It is even implemented in an accessible and easy-to-use computer program, such as EASYANN (http://easyann.gvilella.me/), accessed on 16 February 2021 [32].

To avoid overfitting, the selected data were divided into three parts called the training, test, and prediction sets. Moreover, the number of neurons in the hidden layer was changed in each model from 2 to 50. The Levenberg-Marquardt algorithm [46] was used to determine the parameters needed (weights and bias), with the root mean square error (RMSE) as the target function:

(3) $$RMSE=\sqrt{\frac{1}{N}\sum_{i=1}^{n}{\left(\sigma_{i}^{dataset}-\sigma_{i}^{calc}\right)}^{2}}$$

where $\sigma^{dataset}$ is the value in the dataset and $\sigma^{\mathrm{calc}}$ is calculated by the ANN model.

Apart from RMSE, other statistical parameters were obtained for each model in order to check its accuracy. In particular, the PDs and AADs values, Equations (1) and (2), as well as the coefficient of determination R^2^, were calculated:

(4) $$R^{2}=1-\frac{\sum_{i=1}^{n}{\left(\sigma_{i}^{dataset}-\sigma_{i}^{calc}\right)}^{2}}{\sum_{i=1}^{n}{\left(\sigma_{i}^{dataset}-\bar{\sigma }\right)}^{2}}$$

where $\bar{\sigma }$ represents the mean surface tension value.

Since there are a significant number of properties that could be used as inputs in the ANN model, those most relevant to calculate the surface tension were determined using the so-called factor analysis [47]. This technique investigates connections among variables and tries to find a smaller number of variables or factors explaining those connections. Thus, the factor analysis allows having a clearer view of the data. In particular, the influence of each possible input parameter on the calculated surface tension is analyzed by calculating the so-called “Effect Factor” (EFF). The commercial software package ModeFrontier © was used for this purpose [48].

The following possible factors (i.e., input parameters) were considered: Molecular weight, radius of gyration, critical temperature, reduced temperature (i.e., temperature divided by the critical temperature, T_r_ = T/T_c_), liquid molar volume, boiling temperature (T_b_), critical pressure, acentric factor (ω), and dipole moment. Obviously, the reduced temperature is obligatory, as the surface tension has to take a zero value just when T_r_ = 1, and the others have commonly been used in previous correlation or corresponding-states models (see Table 1) or neural networks.

Results for the EFF values of each input parameter are listed in Table 2. The negative sign indicates that, in general, the surface tension decreases when any of the input parameters under analysis increases. It is also clear that the influence of the radius of gyration, liquid molar volume, and critical pressure is negligible. Therefore, we shall focus attention on the first four parameters listed in Table 2, and then only use them as input parameters in the ANN model. This choice is also reinforced by the fact that other workers adopted these same parameters in previous models [11,12,13,17].

As mentioned above, the database, containing 2051 data, was separated randomly into three subsets, as recommended by Arce et al. [33] and Faúndez et al. [38]: 75% of the data for training, 15% for testing, and the other 10% for prediction. The training set is used to obtain the values of the weights and bias through a back-propagation algorithm. The test set is used to confirm the validity of the model and to avoid overfitting. The prediction set is used to evaluate the accuracy of the model for other new data not included in the design of the model, i.e., the network is blind to the prediction set. Since all the data points are within the database, the network’s extrapolation capacity is not analyzed here, but only its potential for generalization and prediction.

Another important step is the choice of the activation function that determines whether each neuron’s input has to be activated, or not, depending on its relevancy in the predictions given by the model. There are several types of activation functions. With just a linear function, the ANN would be nothing more than a simple regression model. The particular non-linear function used here is the sigmoid function, defined as:

(5) $$s\left(x\right)=\frac{1}{1+e^{x}}$$

Due to its numerous advantages [38], it is a simple function whose output values are from 0 to 1 and has a simple derivative, which ensures fast calculations [49].

In this work, a significant number of ANN architectures and input parameters were considered. In particular, groups of three and four input variables were checked, following the order in Table 2. In a personal computer, the required time to run models including 3–4 variables and 2 to 50 hidden neurons is around 2 or 3 h. The three subsets of data and the initial seed values for the weight and bias were randomly selected in each run. In total, 30 different runs were made for each ANN architecture. Once the values and weight values were determined, the calculations can be made in 3–4 min, depending on the number of variables and hidden neurons.

Finally, in accordance with the suggestions of Faúndez et al. [38] and in view of the results obtained, a configuration with only one hidden layer and only three input parameters (T_r_, T_b_, ω) was chosen. In particular, although in Table 2 the acentric factor and molecular weight have similar EFF values, the inclusion of the latter property did not lead to significantly better results.

**Figure 1:**
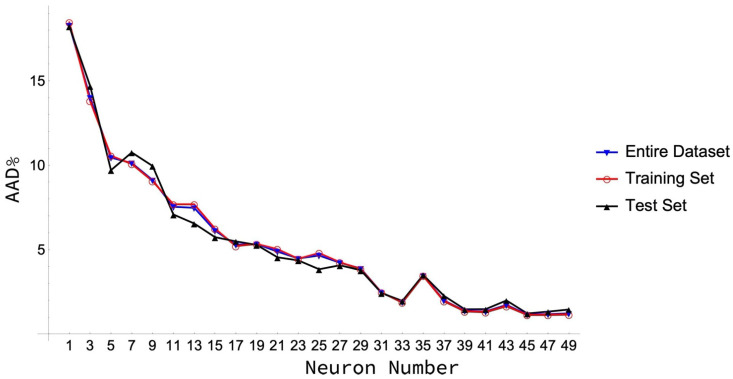
AADs for each dataset as a function of the number of neurons in the hidden layer.

The next step is to determine the appropriate number of neurons that must be placed in each hidden layer. In particular, as shown in Figure 1, a minimum in the AAD values for the training and test sets is reached for 41 neurons. This means that a set of 206 coefficients (weights and biases) is determined in the training process, a number that can be regarded as low [38] when compared to the 2051 surface tension data considered here. This high number of neurons in the hidden layer is needed to obtain a low AAD value with a great majority of data reproduced with a PD below 10%. This low PD is not so easy to reach in the case of the surface tension, as it tends to reach zero as the temperature approaches the critical point.

## 3. Results and Discussion

Table 3 gives detailed results for the architecture of three input variables and one hidden layer that contains 41 neurons. The results for the training, test, prediction, and complete datasets are presented separately. Obviously, the lowest AAD and RMSE values are obtained for the training data set. In any case, for both the test data set (implying that the model is not over-fitted and the calculated weights and biases are adequate) and the prediction data set (implying that new values of the surface tension can be calculated even if they were not considered in the design of the model) the results are very similar.

When the complete data set is considered, the ANN calculates the surface tension with a maximum absolute deviation (PDm) of 14.53%, with an overall AAD of 1.33%. For each of the data sets considered, the RMSE values are very close to zero, and the R^2^ values are all greater than 0.998.

In sum, the selected ANN model consists of an input layer with three neurons associated with the input parameters (T_r_, T_b_, ω), plus one hidden layer containing 41 neurons, and one output neuron with the surface tension for each temperature and fluid. The weight and bias values for each neuron are included in the Supplementary Materials as Table S3.

**Figure 2:**
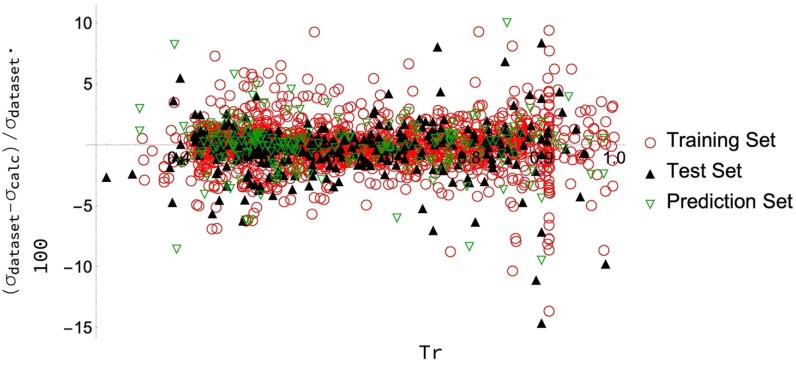
PD (%) for each calculated value with respect to those in each data set versus the reduced temperature, T_r_ = T/T_c_.

It is also important to analyze the behaviour of the PD values for each datum. As seen in Figure 2, the results for the prediction data set are similar to those for the training and test data sets. For the complete data set, only a few data points (indeed, only five out of the 2051 collected) have deviations greater than 10%, and these points mainly correspond to high temperatures. As remarked above, there are two principal reasons behind these greater deviations: (i) At the critical temperature, the surface tension must be null, and then a little absolute deviation between the calculated and database values can produce a very high PD (since the denominator is so small); (ii) for a given fluid, there may be different and not matching values for the same temperature coming from different or even the same sources. The ANN (or any other alternative model) will only give one value so that it is impossible to reduce the deviation of all the points to low values. In the case of slight differences between the values at this temperature, the corresponding PDs will be small, but some cases might occur where this percentage difference is considerable. Some examples will be presented at the end of this section.

Moreover, it is important to consider the separate results for each fluid. Table 4 lists the AAD and PDm values for the 98 acids. The overall AAD is 1.33%, as given in Table 3. In conclusion, the selected ANN gives excellent results for most of the acids considered, leading (as expected) to a general accuracy significantly better than those obtained using corresponding-states models (see Table S2 for details when there is one corresponding-state model with an AAD below 10%).

Of course, the ANN model is not perfect, but, as noted above, the large deviations obtained in some cases can be clearly explained by the behaviour of the data collected for each particular fluid. Several examples will be analyzed in the following paragraphs.

As seen in Table 4, the highest AAD value (5.02%) is obtained for neohexanoic acid, with the PDm of 8.46% and only 11 data are considered. For this fluid, AADs higher than 10% are obtained when the corresponding-states models are used. As Mulero et al. [23] noted, all the values in the DIPPR database for this fluid come from Sugden’s method [10], so that in fact, there are no experimental data available, and only a comparison between predictions is made. For this fluid, therefore, the only conclusion drawn is that different models lead to different values. Moreover, the high PD obtained with the ANN model means that the relationship between the input variables and the surface tension of this fluid is different than that obtained for the other organic acids.

**Figure 3:**
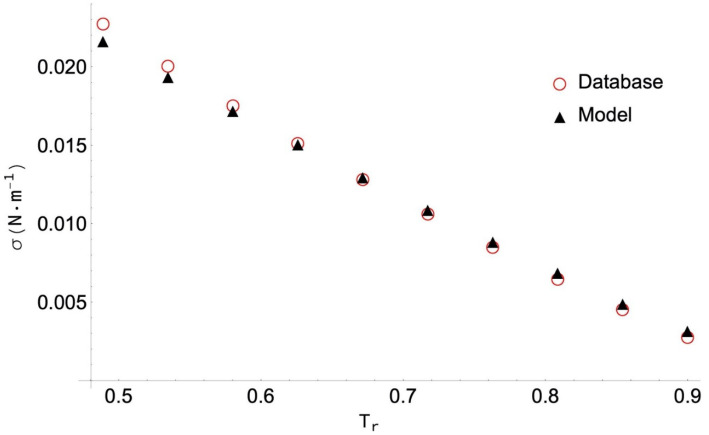
Selected surface tension data for neopentanoic acid and values obtained with the ANN model versus the reduced temperature, T_r_ = T/T_c_.

The highest PDm value (14.53%) is obtained for neopentanoic acid, despite the corresponding AAD below 5%. As in the previous case, the 10 data selected have been obtained by DIPPR with the use of Sugden’s method [10], so no experimental values are included. As shown in Figure 3, the behaviour of the data is homogeneous, and the ANN model reproduces them very well. In this case, the greatest deviations are due to the fact that some of the data is collected located near the critical point, and then the surface tension value is practically zero. In particular, the 14.53% value corresponds to the data at the highest temperature (T_r_ = 0.90), even though the difference between the collected and the calculated values is only 0.0004095 N/m (something that cannot be clearly appreciated in the figure).

**Figure 4:**
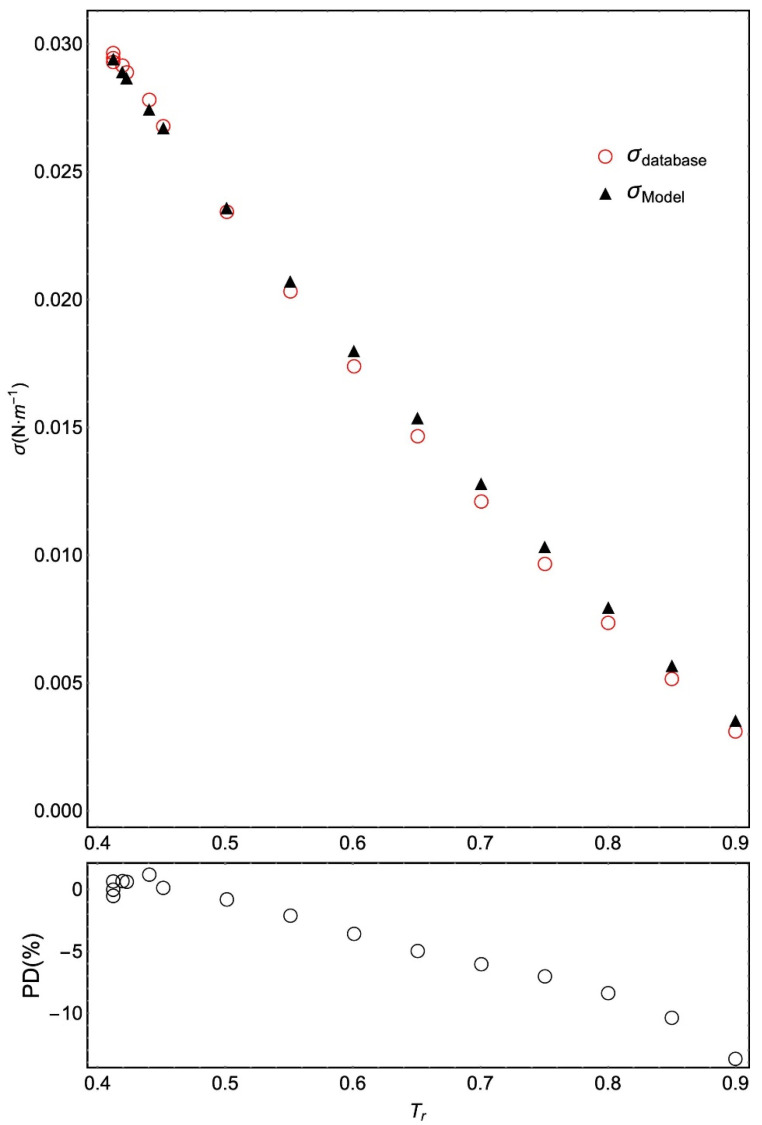
Selected surface tension data for n-nonanoic acid and values obtained with the ANN model versus the reduced temperature, T_r_ = T/T_c_. The absolute values of PD are given below.

In Table 4, a high PDm value (13.58%) can also be seen in the case of n-nonanoic acid. Figure 4 shows the selected data following a regular trend, which is not the same as that obtained with the ANN model. In any case, only for two data points (those at the highest temperatures) does PD exceed 10%. The AAD for this fluid is 3.77% compared with the best corresponding-states model (from Sastri-Rao) in which the AAD is 6.1% (Table S2).

**Figure 5:**
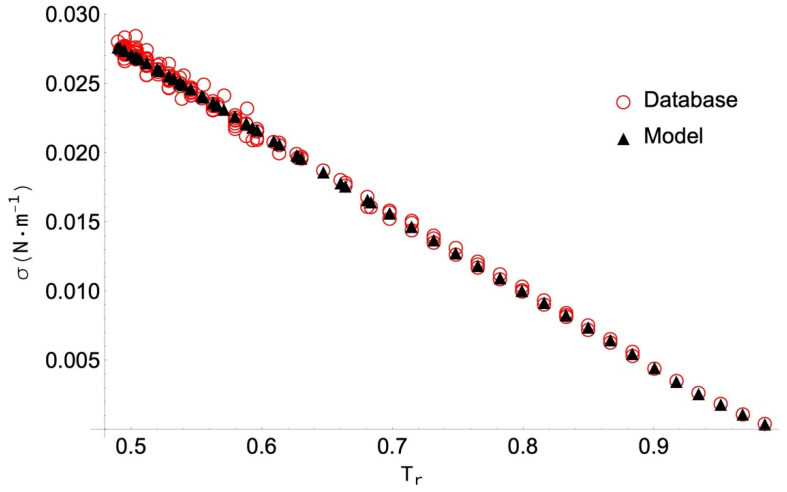
Selected surface tension data for acetic acid and values obtained with the ANN model versus the reduced temperature, T_r_ = T/T_c_.

Let us now consider the case of acetic acid, which is the fluid in which most data were selected. As noted in Mulero et al. [23], the difficulty in achieving good accuracy is due to the extension of the temperature range and the disagreement between some of the data at low temperatures. Thus, that work finally proposed a specific model with four adjustable coefficients, with an AAD of 1.36% and a PDm of 5.28%. As shown in Figure 5, the proposed ANN model reproduces the general trend of the data very well. In Table 4, the present results can be considered as very similar to those given by Mulero et al. [23]. The behaviour of the data and the model for acetic acid is similar to that found in the case of n-butyric and propionic acids, in which 97 and 87 data are available, respectively. Nevertheless, in some temperature ranges, there are no homogeneous values. It is obvious that an ANN or a corresponding-states model can reproduce general behaviour, but not two very different data values at the same temperature.

**Figure 6:**
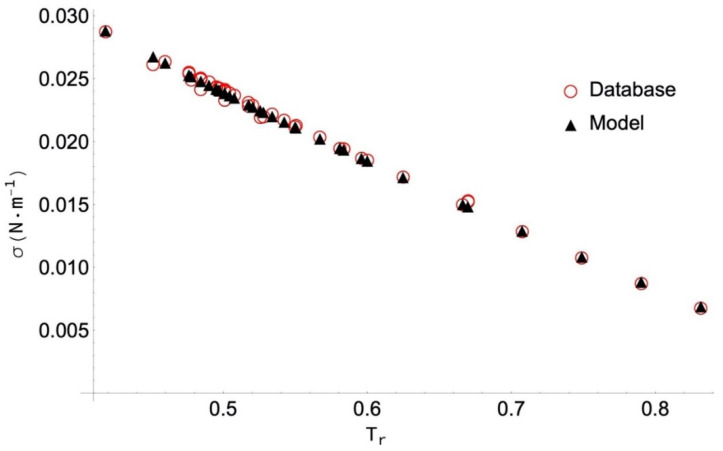
Selected surface tension data for isobutyric acid and values obtained with the ANN model versus the reduced temperature, T_r_ = T/T_c._

As stated before, the general behaviour of the ANN model can be regarded as excellent, since data for 80 fluids are reproduced with AADs below 2%. A clear example of a good result is found in the case of isobutyric acid, in which 46 data are available. Figure 6 shows that, despite the disagreements among the data at low temperatures, the ANN model reproduces the data with a PDm of 2.93% and an AAD of 0.91% which is a very low value.

## 4. Conclusions

In conclusion, it has been shown that a feed-forward multilayer perceptron ANN model with a (3,41,1) architecture can calculate the 2051 surface tension values of 98 liquid organic acids accurately, with an overall AAD of 1.33% and a maximum PD of 14.53% (in the case of neopentanoic acid). Comparatively, the overall AADs in the case of the available corresponding-states models are greater than 20%, although, for a small number of fluids, some particular corresponding-states models can give AADs from 0.7% to 10% (see Table S2).

To train the ANN, and check its predictive capability, the whole data set was randomly split into three parts: 75% for training, 15% for testing or validation, and 10% for prediction. In particular, the 10% data of the prediction set were not considered during the training process and in selecting the best ANN model. In any case, these data are all located within the surface tension and temperature ranges considered, i.e., no extrapolations were made.

Several alternatives were considered for the input properties, and the effect factor was calculated to select those which were most relevant for the surface tension. Different ANN architectures were checked, considering groups of three or four input variables, and in each case, two to 50 neurons were placed in the hidden layer. Each model was run 30 times to find the best results. Finally, the chosen input properties were the reduced temperature, the boiling temperature, and the acentric factor. It has to be mentioned that these input properties are well-known for the fluids studied and are commonly used in the corresponding-states models (although usually with the assistance of some other additional properties). Moreover, the selected model contains 41 neurons in the hidden layer.

Finally, the origin of the disagreements between the collected surface tension values and the calculated ANN models was studied by considering various illustrative examples. Most of the disagreements found are due either to the dispersion of data collected from different sources or to the presence of data close to the critical point. Except for some particular fluids, the collected and calculated data trends are very similar, with the results significantly better than those of other models.

The values obtained here can be used to calculate the surface tension of mixtures using different models that include a dependence on the molar fraction of each component and the values of the surface tension of the pure fluids. On the other hand, a new ANN model could be defined, trained, and validated by collecting data for different mixtures and including the molar fractions directly as input variables or indirectly in the definition of the input properties of the mixture, for instance, defining an acentric factor of the mixture as a function of the molar fractions and acentric factors of the pure components.

## Figures and Tables

**Table 1:** Input properties and results obtained for the considered corresponding-states model and the results obtained. AAD is the absolute average deviation, as defined in Equation (2). N_10_ gives the number of fluids with AAD < 10%. The maximum and minimum AAD values (maxAAD and minAAD), and the overall AAD are also included.

Model	Brock-Bird (1955) [11]	Sastri-Rao (1995) [12]	Pitzer (1995) [13]	Gharagheizi et al. (2013) [17]
Input properties ^a^	P_c_, T_c_, T_r_, T_br_	P_c_, T_b_, T_c_, T_br_, T_r_	P_c_, T_c_, T_r_, ω	T_c_, T, ω, M_w_
N_10_	17	24	10	28
maxAAD (%)	540.3	172.8	947.7	178.6
minAAD (%)	2.2	2.1	0.7	0.8
Overall AAD (%)	48.9	20.2	65.0	31.2

^a^ P_c_: Critical pressure (bar); T_c_: Critical temperature (K); T_b_: Boiling temperature (K); T_r_ = T/T_c_: Reduced temperature; T_br_ = T_b_/T_c_: Reduced boiling temperature; ω: Acentric factor; M_w_: Molecular weight (g/mol).

**Table 2:** Effect factors (EFFs) obtained using the ModeFrontier software [48] ordered by the decreasing absolute value.

Factor	EFF
Reduced Temperature (T_r_ = T/T_c_)	−0.0151
Boiling Temperature (K)	−0.0119
Acentric Factor	−0.0025
Molecular Weight (g·mol^−1^)	−0.0024
Radius of Gyration (m)	−0.0005
Liquid Molar Volume (L·mol^−1^)	−0.0005
Critical Pressure (Pa)	−0.0003

**Table 3:** AADs, maximum absolute deviation (PDm), and root mean square error (RMSE) values for the final artificial neural network (ANN) model.

Dataset	AAD (%)	PDm (%)	RMSE	R^2^
Training	1.28	13.58	0.000387	0.99848
Test	1.47	14.53	0.000408	0.99999
Prediction	1.51	10.10	0.000590	0.99998
Complete	1.33	14.53	0.000425	0.99825

**Table 4:** AAD and PDm values obtained with the selected ANN model.

Fluid	AAD (%)	PDm (%)
1,4-Cyclohexanedicarboxylic acid	1.12	3.46
2,6-Naphthalenedicarboxylic acid	1.14	2.37
2-Ethylbutyric acid	4.29	8.30
2-Ethylhexanoic acid	1.38	2.83
2-Formyl benzoic acid	0.41	1.25
2-Methylbutyric acid	0.86	2.56
2-Methylhexanoic acid	1.05	3.40
2-Methyloctanoic acid	1.84	4.15
4-Hydroxymethyl benzoic acid	0.81	1.20
4-Methoxyphenylacetic acid	0.44	1.18
6-Hydroxyhexanoic acid	0.86	1.73
Abietic acid	1.33	2.42
Acetic acid	1.35	5.25
Acetoacetic acid	0.82	1.57
Acetoxyacetic acid	3.03	7.78
Acetylsalicylic acid	0.37	0.93
Acrylic acid	0.88	3.28
Acryloxy propionic acid	1.10	3.55
Adipic acid	1.58	7.06
Alpha-hydroxyisobutyric acid	0.75	1.95
Ascorbic acid	0.48	1.69
Azelaic acid	0.71	2.24
Benzoic acid	1.57	2.92
Cinnamic acid	2.17	8.59
Cis-crotonic acid	3.18	5.05
Citraconic acid	0.86	3.08
Citric acid	0.53	1.19
Cyclopentylacetic acid	1.63	4.28
Cyclopropane carboxylic acid	4.13	6.83
Dehydroabietic acid	0.75	3.41
Diglycolic acid	0.93	2.07
Dilactic acid	0.28	0.46
Dodecanedioic acid	0.55	1.86
Formic acid	0.80	4.10
Fumaric acid	0.87	2.53
Glutaric acid	1.60	8.69
Glycolic acid	0.80	2.05
Hydroxycaproic acid	1.09	2.03
Ibuprofen	2.05	4.45
Isobutyric acid	0.91	2.93
Isophthalic acid	1.31	2.16
Isopimaric acid	1.45	3.93
Isovaleric acid	0.96	3.29
Itaconic acid	3.40	6.59
Lactic acid	0.68	1.17
Levulinic acid	1.80	4.75
Linoleic acid	1.57	3.07
Linolenic acid	2.84	5.55
Maleic acid	0.91	1.62
Malic acid	0.40	1.40
Malonic acid	0.91	3.31
Methacrylic acid	2.83	4.48
Methoxyacetic acid	1.20	1.94
Monomethyl terephthalate	0.56	0.91
*m*-Toluic acid	1.19	2.89
*n*-Butyric acid	1.49	6.94
*n*-Decanoic acid	3.17	10.10
*n*-Dodecanoic acid	0.86	4.47
*n*-Eicosanic acid	1.83	9.38
Neoabietic acid	1.83	8.56
Neoheptanoic acid	1.67	7.67
Neohexanoic acid	5.02	8.46
Neopentanoic acid	4.65	14.53
*n*-Heptadecanoic acid	1.28	2.51
*n*-Heptanoic acid	0.76	2.07
*n*-Hexadecanoic acid	0.90	1.85
*n*-Hexanoic acid	1.22	5.55
*n*-Nonadecanoic acid	1.63	5.91
*n*-Nonanoic acid	3.77	13.58
*n*-Octadecanoic acid	1.48	3.47
*n*-Octanoic acid	1.63	4.83
*n*-Pentadecanoic acid	0.91	3.88
*n*-Pentanoic acid	0.48	1.35
*n*-Tetradecanoic acid	0.83	2.63
*n*-Tridecanoic acid	1.35	2.87
*n*-undecanoic acid	2.76	7.63
Octahydro-pentalene-1-carboxylic acid	1.64	3.84
Oleic acid	2.42	9.34
*o*-Toluic acid	1.88	5.60
Oxalic acid	0.45	1.96
Palustric acid	2.00	6.30
Peracetic acid	0.22	0.43
Phthalic acid	0.64	1.73
Pimaric acid	1.25	3.93
Pimelic acid	1.83	4.89
Propionic acid	1.11	5.63
*p*-Toluic acid	2.09	3.92
Pyromellitic acid	0.85	2.90
Pyruvic acid	0.72	2.62
Salicylic acid	1.31	2.44
Sebacic acid	0.66	1.59
Suberic acid	0.84	3.27
Succinic acid	1.04	9.67
Tartaric acid	0.63	1.15
Tetradecanedioic acid	0.89	1.99
Trans-crotonic acid	4.26	11.00
Trilactic acid	2.83	5.58
Trimellitic acid	0.63	1.72

## Data Availability

The data presented in this study are available in Supplementary Materials.

## Supplementary Materials

The following are available online. Table S1: Names of liquids, with their respective numbers of data, temperature range, and input property values; Table S2: AAD values for fluids in which at least one of the corresponding-states models studied gives a value below 10%. The AADs obtained from the ANN model are also given for comparison; Table S3: Values of the bias and weights for the ANN model. Results for particular fluids can be obtained from authors upon request.

## Sample Availability

Samples of results obtained for the compounds are available from the authors.
