# Supplementary material for: Surface Tension of Liquid Organic Acids: An Artificial Neural Network Model
Source: Molecules. 2021 Mar 15;26(6):1636. doi: 10.3390/molecules26061636 (PMC7998689; doi:10.3390/molecules26061636)
Supplement: Supplementary file 1 [file molecules-26-01636-s001.pdf]

Surface Tension of Liquid Organic Acids: an Artificial Neural Network Model

M. Pierantozzi[1], A. Mulero[2], and I. Cachadiña[2]*

SUPPLEMENTARY MATERIAL

Table S1. List of fluids, number of data, temperature range, and values of input properties (critical temperature, acentric factor, and boiling temperature). The values for these three fixed properties were selected values in the DIPPR database [27].

Fluid	N	T range (K)	Tc (K)	ω	Tb (K)
1,4-Cyclohexanedicarboxylic acid	16	583–737.82	819.8	1.00497	611.60
2,6-Naphthalenedicarboxylic acid	11	640.15–813.6	904	1.16013	695.00
2-Ethylbutyric acid	11	258.15–588.15	655	0.632579	466.95
2-Ethylhexanoic acid	16	235–607.14	674.6	0.801289	500.66
2-Formyl benzoic acid	11	370.15–694.8	772	0.818818	561.00
2-Methylbutyric acid	11	193–573	643	0.589443	450.15
2-Methylhexanoic acid	15	230–599.4	666	0.695679	483.00
2-Methyloctanoic acid	14	240–661.57	694	0.791271	518.15
4-Hydroxymethyl benzoic acid	12	454.65–732.96	814.4	1.22799	614.70
4-Methoxyphenylacetic acid	11	356.15–691.2	768	0.898421	564.00
6-Hydroxyhexanoic acid	11	313.15–657	730	1.17757	557.00
Abietic acid	11	446.65–646.65	832	1.05285	649.70
Acetic acid	165	290.1–583.15	591.95	0.466521	391.05
Acetoacetic acid	15	310.65–610.65	678.5	0.707335	478.20
Acetoxyacetic acid	15	363.8–687.89	764	0.710447	542.00
Acetylsalicylic acid	11	408.15–688.5	765	0.832697	564.00
Acrylic acid	23	286.15–546.15	615	0.538324	414.15
Acryloxy propionic acid	15	283.8–656.55	729.5	0.801762	533.80
Adipic acid	16	426.55–756.9	841	0.777756	609.00
Alpha-hydroxyisobutyric acid	11	352.15–597.6	664	0.983288	485.15
Ascorbic acid	11	465.15–695.15	783	2.38853	637.00
Azelaic acid	39	381.25–832.13	844	0.89229	632.90
Benzoic acid	22	395.45–675.45	751	0.602794	522.40
Cinnamic acid	38	406.15–776.96	797	0.712052	573.15
Cis-crotonic acid	6	288.65–433.65	647	0.57228	445.05

Citraconic acid	11	356.15–736.15	829	0.926873	607.00
Citric acid	15	427.1–720.18	800.2	2.04824	640.80
Cyclopentylacetic acid	11	286.65–637.2	708	0.614415	501.15
Cyclopropane carboxylic acid	4	295.55–359.05	671	0.604847	456.15
Dehydroabietic acid	11	445.65–745.2	828	0.91535	645.00
Diglycolic acid	11	421.15–731.15	820	1.08065	610.00
Dilactic acid	11	385.65–594	660	0.906812	489.00
Dodecanedioic acid	16	401.15–762.3	847	1.04179	658.10
Formic acid	74	282.35–483.15	588	0.312521	373.71
Fumaric acid	10	573.15–690.15	771	0.988463	563.15
Glutaric acid	50	371.65–827.99	840	0.752616	600.00
Glycolic acid	11	352.65–552.65	616	1.05917	443.00
Hydroxycaproic acid	11	334–614.7	683	1.13754	518.00
Ibuprofen	11	348.62–688.5	765	1.01014	580.45
Isobutyric acid	46	253.15–503.15	605	0.61405	427.65
Isophthalic acid	11	619.15–899.15	1007	1.06133	753.00
Isopimaric acid	20	435.65–726.39	807.1	0.790828	623.10
Isovaleric acid	35	273.15–573.15	629.09	0.681753	449.68
Itaconic acid	11	438.75–738.75	821	0.924722	601.00
Lactic acid	11	291.15–561.15	675	1.0221	490.00
Levulinic acid	11	313.15–388.15	738	0.755749	530.00
Linoleic acid	11	268.15–688.15	775	1.1801	628.00
Linolenic acid	11	262.05–692.05	780	1.18697	632.00
Maleic acid	11	403.8–695.7	773	0.997587	565.00
Malic acid	11	403.15–693.15	781	1.52561	602.00
Malonic acid	39	409.15–823.11	834	0.738273	580.00
Methacrylic acid	11	288.15–595.8	662	0.331817	434.15
Methoxyacetic acid	11	281–621	691	0.624846	478.26
Monomethyl terephthalate	16	492.54–718.02	797.8	0.876982	593.30
m-Toluic acid	6	413.15–473.15	754	0.680936	536.15
n-Butyric acid	97	273.15–523.15	615.7	0.675003	436.42
n-Decanoic acid	28	304.55–649.89	722.1	0.813724	543.15
n-Dodecanoic acid	38	316.98–668.7	743	0.89828	571.85
n-Eicosanic acid	11	348.4–738	820	1.08228	668.53
Neoabietic acid	11	446.4–730.8	812	0.859767	631.00
Neoheptanoic acid	11	230.-595.8	662	0.635379	475.65
Neohexanoic acid	11	259.15–587.7	653	0.567133	460.15
Neopentanoic acid	10	309.08–568.8	632	0.512805	436.95
n-Heptadecanoic acid	20	334.25–675.75	792	1.01866	634.65
n-Heptanoic acid	30	273.15–573.15	677.3	0.759934	496.15
n-Hexadecanoic acid	26	335.9–423.15	785	0.991904	623.15
n-Hexanoic acid	40	270.15–593.19	660.2	0.733019	478.85
n-Nonadecanoic acid	20	341.23–729.9	811	1.06769	657.15
n-Nonanoic acid	16	293.15–639.63	710.7	0.778706	528.75
n-Octadecanoic acid	29	342.75–685.51	803	1.03936	647.15

n-Octanoic acid	32	289.65–624.83	694.26	0.773427	512.85
n-Pentadecanoic acid	16	325.68–697.5	774	0.963881	610.65
n-Pentanoic acid	36	253.15–503.15	639.16	0.706632	458.95
n-Tetradecanoic acid	24	327.4–422.45	763	0.953534	599.35
n-Tridecanoic acid	14	315.01–678.6	754	0.913368	585.25
N-undecanoic acid	13	301.63–658.8	732	0.85309	557.15
Octahydro-pentalene-1-carboxylic acid	16	312.9–719.55	799.5	0.69285	576.90
Oleic acid	17	293.15–453.15	781	1.18222	633.00
o-Toluic acid	7	413.15–473.15	751	0.657237	532.00
Oxalic acid	39	462.65–818.63	828	0.286278	516.00
Palustric acid	19	441.65–770.72	789	0.917368	616.00
Peracetic acid	11	272.95–492.95	552	0.752294	383.00
Phthalic acid	11	464.15–714.15	800	1.05849	598.00
Pimaric acid	20	491.65–726.39	807.1	0.791651	623.10
Pimelic acid	20	378.15–830.11	842	0.811396	617.30
Propionic acid	87	252.75–573.15	600.81	0.579579	414.32
p-Toluic acid	16	452.34–697.14	775	0.629104	547.65
Pyromellitic acid	11	554–794	893	1.83014	722.00
Pyruvic acid	11	286.75–566.75	634.52	0.670904	438.15
Salicylic acid	11	431.75–661.75	739	0.851182	529.00
Sebacic acid	39	406.25–833.75	845	0.960796	642.10
Suberic acid	20	416.45–832.06	843	0.85142	624.80
Succinic acid	39	460.85–828.33	838	0.743044	591.00
Tartaric acid	11	479.15–739.15	828	2.01095	660.00
Tetradecanedioic acid	16	399.15–764.1	849	1.1671	674.50
Trans-crotonic acid	11	344.55–594.55	666	0.570794	458.15
Trilactic acid	10	312.15–660.58	777	1.42514	619.00
Trimellitic acid	10	511.15–747.71	860	1.49114	675.00

Table S2. AAD (%) values for those fluids for which at least one of the studied corresponding-states models (CSM) gives a value below 10% (BB = Brock- Bird [11]; SR = Sastri-Rao [12]; PZ = Pitzer [13]; Gh = Gharagheizi *et al.* [17]). AADs obtained from the ANN model are also given for comparison.

Fluid	AAD(%) CSM	AAD(%) ANN
1,4-Cyclohexanedicarboxylic acid	4.2 Gh	1.12
2,6-Naphthalenedicarboxylic acid	3.2 Gh	1.14
2-Ethylbutyric acid	8.3 BB<br>2.8 PZ	4.29
2-Ethylhexanoic acid	9.5 SR	1.38
2-Methylbutyric acid	3.1 SR	0.86
2-Methylhexanoic acid	8.8 SR	1.05
4-Hydroxymethyl benzoic acid	6.4 Gh	0.81
Acetic acid	6.2 SR	1.35
Acetoxyacetic acid	8.0 BB<br>3.1 PZ	3.03
Acetylsalicylic acid	2.4 BB<br>6.9 PZ	0.37
Acrylic acid	2.9 SR	0.88
Acryloxy propionic acid	1.9 Gh	1.10
Adipic acid	3.3 SR	1.58
Benzoic acid	2.5 Gh	1.57
Cinnamic acid	6.0 Gh	2.17
Cis-crotonic acid	8.5 SR	3.18
Citraconic acid	6.2 Gh	0.86
Cyclopropane carboxylic acid	3.1 SR	4.13
Dehydroabietic acid	3.8 BB<br>8.7 PZ	0.75
Dilactic acid	3.8 SR<br>6.7 Gh	0.28
Dodecanedioic acid	7.2 Gh	0.55
Formic acid	6.3 BB<br>4.5 PZ	0.80
Fumaric acid	8.1 Gh	0.87
Glycolic acid	4.3 Gh	0.80
Hydroxycaproic acid	9.1 SR	1.09
Ibuprofen	3.2 SR<br>8.2 Gh	2.05
Isobutyric acid	4.8 SR	0.91
Isophthalic acid	2.6 PZ	1.31
Isovaleric acid	2.1 SR	0.96
Itaconic acid	4.7 Gh	3.40
Linoleic acid	7.0 BB	1.57
Linolenic acid	4.5 PZ	2.84

Methacrylic acid	3.4 SR	2.83
Monomethyl terephthalate	9.1 PZ	0.56
m-Toluic acid	6.9 Gh	1.19
n-Butyric acid	3.9 SR	1.49
n-Dodecanoic acid	9.0 Gh	0.86
n-Eicosanic acid	2.3 BB<br>9.8 Gh	1.83
Neoabietic acid	1.4 PZ	1.83
Neoheptanoic acid	8.5 SR	1.67
Neopentanoic acid	9.3 SR	4.65
n-Heptadecanoic acid	4.0 BB<br>1.8 Gh	1.28
n-Heptanoic acid	5.7 SR	0.76
n-Hexadecanoic acid	5.9 BB<br>0.8 Gh	0.90
n-Hexanoic acid	6.7 SR	1.22
n-Nonadecanoic acid	2.6 BB<br>6.1 Gh	1.63
n-Nonanoic acid	6.1 SR	3.77
n-Octadecanoic acid	3.5 BB<br>3.9 Gh	1.48
n-Octanoic acid	6.4 SR	1.63
n-Pentadecanoic acid	6.1 BB<br>2.9 Gh	0.91
n-Pentanoic acid	4.1 SR	0.48
n-Tetradecanoic acid	9.4 BB<br>3.4 Gh	0.83
n-Tridecanoic acid	5.1 Gh	1.35
Octahydro-pentalene-1-carboxylic acid	2.2 BB<br>7.9 PZ	1.64
Oleic acid	5.7 BB<br>7.7 Gh	2.42
o-Toluic acid	8.0 Gh	1.88
Phthalic acid	3.8 Gh	0.64
Propionic acid	4.0 SR	1.11
p-Toluic acid	5.3 BB<br>0.7 PZ	2.09
Pyromellitic acid	9.8 SR	0.85
Pyruvic acid	9.4 BB	0.72
Tetradecanedioic acid	6.2 Gh	0.89

Table S3. Bias and weight values for the ANN model.

Neuron Number		Reduced temperature	Boiling Temperature	Acentric Factor	Output neuron
	bias	Weights			
1	−19.14	−0.10	26.53	−1.41	1.67
2	234.33	−0.02	−160.96	−245.39	−26.04
3	162.18	−0.70	−187.98	−22.16	7.14
4	22.87	0.10	−0.44	−54.68	−24.29
5	15.43	−12.31	−3.86	0.42	34.86
6	−24.59	0.04	23.00	19.13	1.50
7	120.23	1.06	−85.55	−135.34	1.65
8	35.95	−0.51	−83.17	41.35	37.17
9	−1.28	0.55	5.80	−13.92	43.69
10	20.10	0.30	−21.75	−12.74	−89.88
11	−3.43	0.32	7.49	−10.25	−136.22
12	42.19	0.11	−42.06	−15.85	53.20
13	−100.41	−0.24	100.49	45.99	33.39
14	48.20	−48.80	5.17	1.28	2.53
15	−31.50	−3.72	35.39	22.08	42.16
16	−53.23	0.04	−28.29	95.33	−17.84
17	−16.18	−0.22	35.30	−7.02	−108.47
18	−52.01	−0.11	54.15	12.93	−10.59
19	−24.00	18.84	−22.59	7.31	−75.86
20	−19.26	−0.03	−9.10	34.42	−61.06
21	45.34	0.01	−16.49	−52.00	−24.54
22	−10.52	−0.55	107.46	−137.70	74.33
23	−53.66	−0.26	26.82	46.01	24.66
24	−86.12	−0.32	89.06	43.25	95.71
25	96.34	0.10	−60.69	−68.87	47.22
26	12.27	−8.63	−2.14	−0.22	−53.48
27	49.01	0.08	−54.77	−2.76	11.20
28	16.87	−14.44	−4.01	1.08	−10.96
29	−2.88	39.07	−34.57	−23.09	−0.96
30	−28.99	5.85	26.80	15.88	−62.88
31	−170.73	0.00	77.06	180.45	−34.22
32	9.08	−0.29	22.05	−56.33	46.80

33	−119.36	−0.01	61.04	114.44	−45.86
34	8.79	0.31	−17.21	1.46	−25.73
35	−8.32	0.37	−27.99	64.20	−22.14
36	−180.40	0.85	209.22	23.93	−25.14
37	8.34	0.54	−105.81	139.91	−11.14
38	2.25	−38.50	34.67	23.29	5.59
39	−23.44	−0.58	10.85	80.29	−24.84
40	29.85	−0.13	−38.35	6.36	188.56
41	38.73	0.08	−39.16	−16.99	−7.22
Output	31.37				
